# S100 proteins as a key immunoregulatory mechanism for NLRP3 inflammasome

**DOI:** 10.3389/fimmu.2025.1663547

**Published:** 2025-08-28

**Authors:** Shahinur Acter, Qing Lin

**Affiliations:** Department of Anesthesiology and Critical Care Medicine, Johns Hopkins University School of Medicine, Baltimore, MD, United States

**Keywords:** S100A8, S100A9, DAMP, inflammasome, gasdermin D, pulmonary hypertension

## Abstract

The S100 superfamily of proteins consists of Ca2+-binding proteins characterized by the EF-hand motif. Certain members of this protein family, such as S100A8, S100A9, and S100A12, have been effectively utilized as biomarkers for the detection and evaluation of prognosis in immunological diseases. These proteins are also identified as damage-associated molecular pattern (DAMP) molecules, which exhibit significant upregulation in various autoimmune disorders, cancers, and neurodegenerative diseases. Following tissue injury, necrotic or immune cells release or secrete DAMPs to initiate inflammatory responses. This signaling further creates autocrine and paracrine positive feedback loops that amplify and sustain the inflammatory response. The NLRP3 inflammasome pathway is a pivotal component in these DAMP-induced immune regulatory mechanisms. This review summarizes the regulatory roles of S100 protein family in NLRP3 inflammasome signaling and their functions in innate and adaptive immunity, with an emphasis on pulmonary hypertension. Moreover, we examine the interactive feedback mechanisms among NLRP3 inflammasome, S100A8/A9, and Gasdermin D, exploring their implications in autoimmune diseases.

## 1 Introduction

An inflammasome is a protein complex consisting of three parts: a sensor protein, an adaptor, and pro-caspase-1 (1). It is an important part of the innate immune system, also known as the body’s defense against pathogens and tissue damage (2). In recent years, the NOD-like receptors (NLR) family has been instrumental in inflammatory responses and has attracted enormous research attention from scientists in the field of immunology research (3). NLR family are subdivided into four subfamilies: NLRA, NLRB, NLRC, and NLRP, These subfamilies have been found to promote inflammasome assembly and inflammatory response by activating multiple downstream signals (4). Among these, NLRP3 (NOD-like receptor protein 3) has gained extra research attention given its in-depth link to the innate immune system; in particular, it is expressed predominantly in macrophages, neutrophils, and dendritic cells as a component of the inflammasome (4). It is an important innate sensor of structurally diverse metabolic damage-associated molecular pattern molecules (DAMPs) (1). This NLRP3 complex protein mediates caspase-1 activation, followed by the secretion of proinflammatory cytokines IL-1/IL-18 in response to threats such as microbial infection and cellular damage. It is also known as apoptosis-associated speck-like protein (5). Regulating the NLPR3 inflammasome is essential due to its crucial role in the immune response. Increasing evidence suggests that the dysregulated condition of NLPR3 inflammasome can result in excessive inflammation, which contributes to the development of chronic inflammatory diseases, including autoimmune disorders, metabolic diseases, cardiovascular diseases, and cancer (6, 7). Therefore, a clear understanding of the mechanism behind NLPR3 response is crucial in order to maintain desirable immune function and tissue homeostasis.

**Figure 1:**
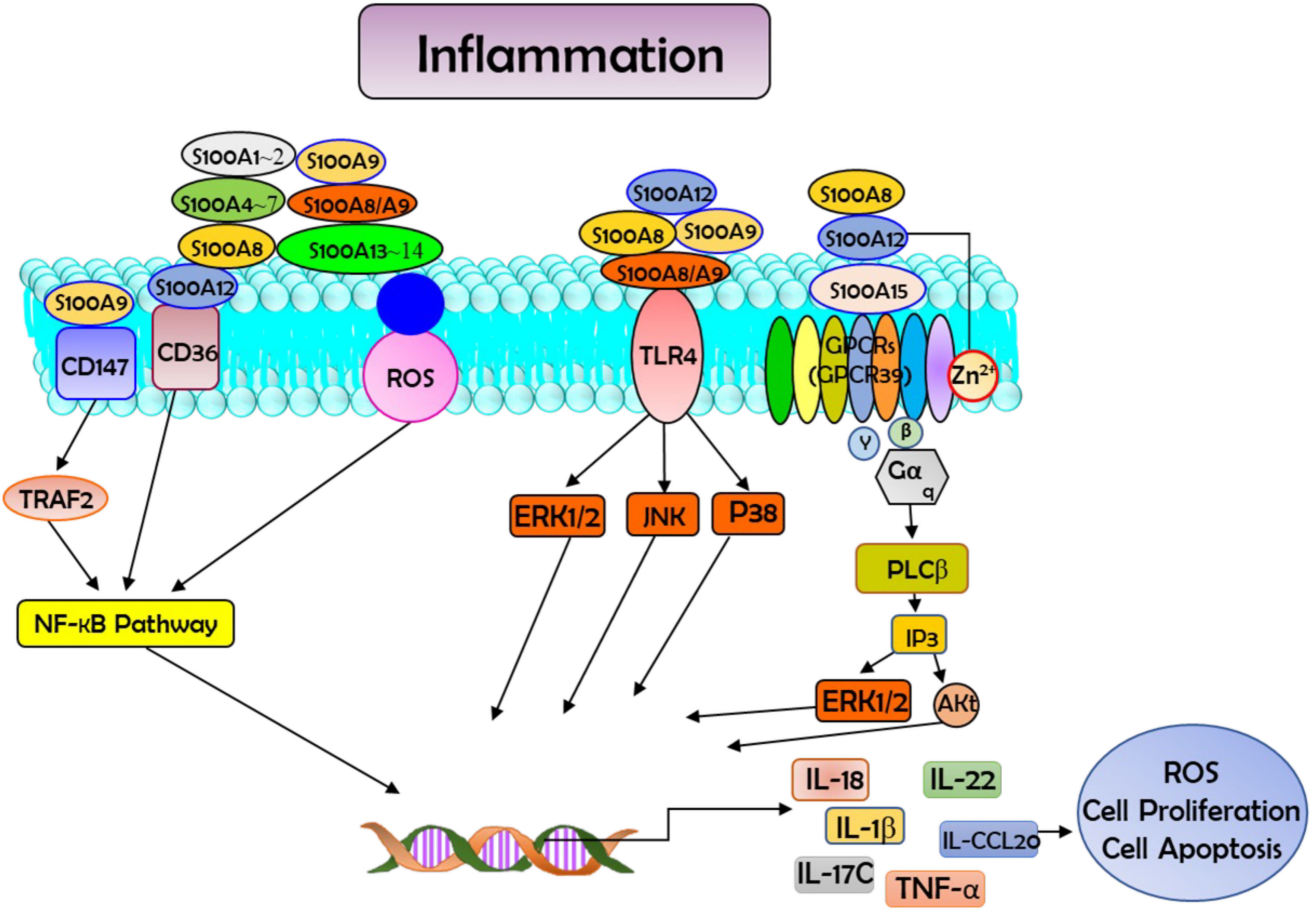
Schematic diagram showing the interaction between S100 proteins and their receptors in the cells. Such interactions activate the downstream signal transductions and regulate multiple cellular processes, such as reactive oxygen species (ROS) production, apoptosis, and cell proliferation. In this process they participate in the modulation of inflammation and have vital roles in combating microbial infection through zinc-mediated nutritional immunity. Akt=protein kinase B; CCL20, ERK1/2=fibroblast growth factor receptors; GPCRs, G protein coupled receptors (GPR39 is used as an example); IL-18, interleukin-18; IP3, Inositol Trisphosphate; NF-κB, nuclear factor kappa B; P38, p38 MAP-Kinases; PI3K, phosphatidylinositol 3-kinase; PLCβ, Phospholipase C β; RAGE, receptor for advanced glycation end products; STAT, Signal Transducer and Activator of Transcription; TLR4, Toll-like receptor 4; TNF-α, tumor necrosis factor-α; TRAF2, tumor necrosis factor receptor-associated factor 2.

Growing evidence suggests that S100 protein family proteins play a significant role as mediators in the initiation and maintenance of inflammation (8, 9). These proteins play a dual role within inflammatory networks: they act as inflammation-responsive proteins but can also amplify the inflammatory signal which produced them in the first place (8). As a family of calcium-binding cytosolic proteins with multifunction properties, S100s have an extensive range of intracellular and extracellular functions through regulating calcium balance. The intracellular functions of this protein family involve interaction with intracellular receptors (8, 9). This protein family plays regulatory roles in multiple cellular processes, including apoptosis, proliferation, differentiation, migration and invasion, energy metabolism, calcium homeostasis, and protein phosphorylation (shown in **Figure 1**) in a number of cells types, including monocytes, macrophages, neutrophils, lymphocytes, myoblast, epithelial cells, endothelial cells, smooth muscle cells, neurons, and fibroblasts. Moreover, S100 proteins have demonstrated their significant role in governing immune homeostasis, post-traumatic injury, and inflammation (10). There are some members of these S100 proteins that function as DAMP molecules that could be released from the cells upon stress or activation of phagocytes such as neutrophils and macrophages (10). DAMPs are a series of intracellular molecules that are linked with apoptosis and tissue damage via instigating a rapid inflammatory response (11). Studies suggest that by binding to the pattern receptors such as Toll-like receptors (TLRs) and receptors for advanced glycation end products (RAGE), S100 proteins actualized as threat signals leading to the activation of immune cells and endothelial cells (12). S100A8 is a calcium-binding protein belonging to the S100 protein family. The S100A8 protein plays a key role in stimulating the NLPR3 inflammasome, which has been identified as a pro-inflammatory mediator, by triggering the activation of this immune complex, resulting in the release of inflammatory cytokines like pro-interleukin-1β (IL-1β) (13, 14).

In this review article, we provide a comprehensive and detailed overview of the NLPR3 inflammasome and S100 protein family and the biological functions of DAMP molecules (S100A8/A9) and highlight their multifunctional role during immune response, with a major focus on their role in inflammatory conditions.

## 2 S100A protein family and their functions

### 2.1 S100 protein family proteins function as immune markers

**Figure 2:**
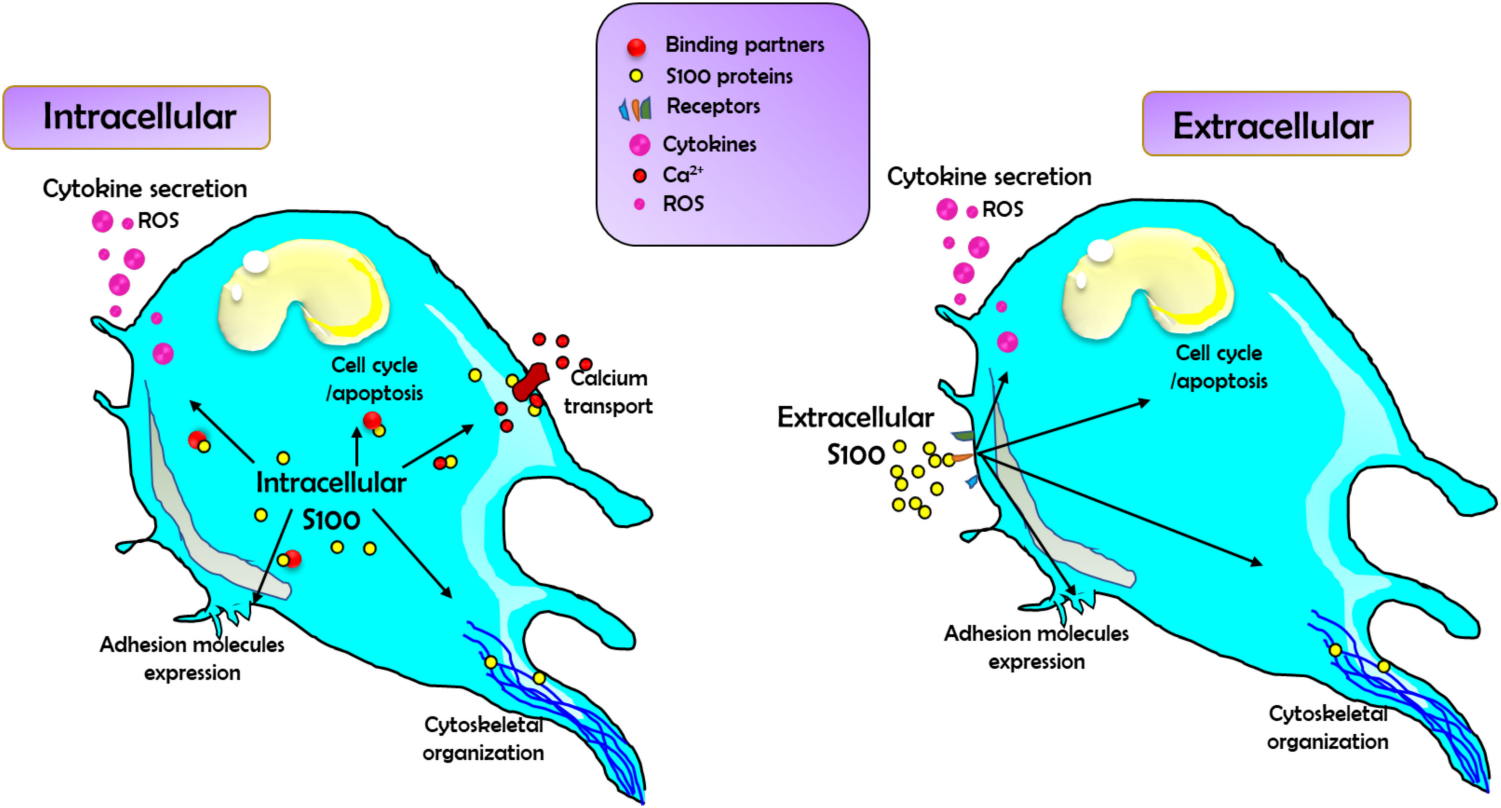
The schematic diagram demonstrates the summary of the general roles of S100 family proteins in regulating cell function. Left: S100 family proteins act intracellularly by binding to and modulating the function of a wide range of binding partners, including those involved in regulating cytokine and reactive oxygen species (ROS) production and release, calcium transport (both through directly binding Ca^2+^ channels and Ca^2+^ regulators), cell cycle, apoptosis, and adhesion molecule expression, as well binding to cytoskeletal components. Right: Secreted S100 proteins can also act as DAMPs to signal via an array of cell surface receptors. This binding will activate signaling pathways to affect changes in cytokine/ROS production, cell cycle, apoptosis, adhesion molecule expression, and changes in cytoskeletal reorganization and migration.

The S100A subfamily is a group of the superfamily of Ca^2+^-binding proteins, which is characterized by the specific Ca^2+^-binding motif, the elongation factor hand (EF-hand) (15). In 1965, S100 proteins S100A1 and S100B were first extracted as neural proteins from the bovine brain and were named S100 due to their solubility in 100% saturated ammonium sulfate (16). There are at least 24 different small acidic proteins (10-12k Da) in this protein family, and they each share 25-65% similarity in their amino acid sequence (16). Among the S100 protein family, S100A8 (MRP8, calgranulin A) and S100A9 (MRP14, calgranulin B) are calcium-binding proteins expressed in cells of myeloid origin (12). This protein family is found as anti-parallel homo- and heterodimers, with each monomer consisting of two helix-loop-helix EF-hands (EF-1 and EF-2) that are connected by a hinge region and flanked by conserved hydrophobic residues at the C- and N-terminal ends (17). Over the years, 3D structures of S100 proteins have been revealed in three different forms that include bound to Ca^2+^, bound to its target protein, or in its apo (Ca^2+^free) form (18). Studies suggest that certain S100 members have been described to bind to the same target molecules (19). For example, S100A1, S100A6, and S100B interact with annexin A6, while S100A1, S100A2, S100A4, and S100B bind to the tumor p53 (19). Studies on the structure of the S100-target complexes have revealed that S100 protein family members apply different mechanisms for target recognition despite the similar conformational change induced by Ca^2+^ binding in all S100 protein family members (20). Moreover, the region exposed upon Ca^2+^ binding comprises the most variable portions of the S100 sequences (hinge and C-terminal regions) that are found enough to discriminate against different target proteins (18, 20). Additionally, the distribution of hydrophobic and charged residues, together with differences in surface configurations, contributes to the specific target binding patterns described among all the members of the S100 protein family (18, 20). The roles of the S100 protein family are summarized in **Figure 2**.

Studies show that extracellular S100 proteins are involved in the activation of G protein-coupled receptors, such as heparan sulfate proteoglycans or N-Glycans and scavenger receptors in autocrine and paracrine manners (15). S100A proteins can be detected in biological fluids, including urine, cerebrospinal fluid, serum, sputum, and feces. Therefore, extracellular S100 proteins are considered biomarkers that are associated with certain diseases (9, 15). It has been demonstrated that certain members of the S100 proteins family, including S100A12, S100A8/A9, and S100B, are linked to specific diseases like autoinflammatory diseases, stroke, and trauma (9). Oesterle et al. have successfully detected an increased amount of S100A12 in the blood of patients with diabetes, and they found that this increase correlated with a higher risk of the development of cardiovascular disease (21). Another research group, Bogdanova et al., detected an increased amount of serum concentration of S100A12 and other acute-phase inflammatory markers after investigating 35 patients with periodic disease (PD), and they found that the level of S100A12 in PD was significantly higher in comparison to other familial periodic fevers (22). S100A12 was more sensitive to the subclinical activity of autoinflammatory diseases when compared to other inflammatory biomarkers, such as neutrophil counts, fibrinogen, C-reactive protein (CRP), and erythrocyte sedimentation rate (22). A study done by another group has demonstrated similar findings, where upregulated concentrations of S100A12 in serum were observed in patients with Familial Mediterranean fever in comparison to controls, which suggests this protein is a novel biomarker (23).

In various studies, S100A8 and S100A9 have been identified as novel diagnostic markers to aid in differential diagnosis (24, 25). Recently, it has been shown that the expression of S100A8/A9 was high in human atherosclerotic lesions, and the blood levels were also on the rise among the patients with coronary artery diseases (CAD), suggesting implied S100A8/A9 might act as a biomarker for cardiovascular events (26). Similar findings were demonstrated by Xia et al., where serum S100A8/A9 levels were elevated in 178 CAD patients with unstable angina pectoris or acute myocardial infarction, and the level of S100A8/A9 was significantly positively linked with CRP (27). These studies represent S100A8/A9 as a novel biomarker for the CAD (28). The serum levels of S100A8/A9 were dramatically increased in IL-1Ra^−/−^ mice, contributing to bone erosion, cartilage damage, and synovial inflammation (21). Therefore, S100A8/A9 is considered a systemic or local biomarker to evaluate the extent of inflammation and inflammatory joint destruction in seronegative arthritis (21). The S100A8/A9 protein complex plays a crucial role as a mediator in the initiation and maintenance of inflammation and has been applied as a valuable clinical biomarker for therapeutic response monitoring (29). Additionally, it was identified as an interesting biomarker to monitor disease activity in chronic inflammatory disorders, including inflammatory bowel disease and rheumatoid arthritis (29).

Increasing evidence suggests that S100 proteins are secreted from cells and exert cytokine-like functions through the binding and activation of cell surface receptors such as the RAGE, TLR-4, ErbB4 receptor, the dopamine D2 receptor, amyloid-β, and annexins (29). It has been demonstrated that various S100 proteins contribute to leukocyte migration (9). For example, S100A8/A9 has been reported to induce pro-inflammatory cytokine production in macrophages and nucleophiles through the activation of the nuclear factor-kB (NF-κB) and p38 mitogen-activated protein kinase pathways and mediate immune cell migration (9, 30). Moreover, the evidence shows that S100A12 has an impact in inducing the production of pro-inflammatory cytokines interleukin (IL) -6 and -8 through RAGE-dependent NF-κB activation, which results in the recruitment of monocytes (9). Additionally, S100A10 protein has been reported to recruit macrophages to tumor sites, while S100A8/S100A9 have been shown to signal through RAGE to mediate the effect of tumor necrosis factor (TNF)-α on the differentiation of myeloid-derived suppressor cells, which demonstrates their regulatory involvement in immune responses (9, 31). The roles and associations of S100A proteins with NLRP3 inflammasome signaling are complex, involving various cellular activation processes observed across multiple studies. **Table 1** summarizes the effects of different S100 family proteins on NLRP3 regulation and related diseases (32–35).

**Table 1:** Summary of the roles of S100 family proteins in NLRP3 regulation, their immunoregulatory mechanisms, and associated diseases.

Members of S100 protein family	Types of cells activated	Regulation process of the NLRP3 inflammasome	Associated diseases
S100A8/A9 (Calprotectin)	Neutrophils, Monocytes/Macrophages, Dendritic cells	Bind TLR4/RAGE → NF-κB↑ → NLRP3↑ → pro-IL-1β transcription↑	Rheumatoid arthritis, atherosclerosis, inflammatory bowel disease, type 2 diabetes, myocardial infarction
S100A12	Neutrophils	Bind RAGE → 1) NF-κB↑ → transcriptional upregulation of inflammasome components; 2) ATP release → NLRP3↑ → pyroptosis	Vasculitis, allergic inflammation, coronary artery disease
S100A4	Fibroblasts, Macrophages, Dendritic Cells, T-lymphocytes.	Bind RAGE → NF-κB↑ → primes NLRP3	Tumor progression, and autoimmune diseases, including psoriasis, rheumatoid arthritis, and neurodegeneration.
S100B	Astrocytes, Schwann Cells	Bind RAGE→ NF-κB↑ → NLRP3 & pro-cytokines↑	Alzheimer’s disease, multiple sclerosis

### 2.2 Expression and distribution of S100A8/S100A9 proteins

Recent studies show that among the S100 protein family, S100A8 and S100A9 are specifically linked to innate immune function through their expression in immune cells of the myeloid lineage (36, 37). S100A8/A9 proteins are mainly derived from myeloid cells such as monocytes, neutrophils, and macrophages, with constitutive expression believed to be limited to neutrophils and monocytes (29). S100A8/A9 proteins comprise 93 and 113 amino acids with molecular weights of 10.8 and 13.2 kDa, respectively (29, 38). S100A8/A9 proteins comprise approximately 45% of the cytoplasmic proteins in neutrophils, and only 5% are constitutively expressed in monocytes (29, 38). These proteins are found in various structures, including homodimers, heterodimers, and tetramers (29, 39). The homodimer is less stable, leading to a preference for the formation of noncovalently bonded complexes, and this is the predominant form of S100A8/A9 in physiological settings (40). After reaching Ca^2+^ concentration at a certain threshold, S100A8/A9 heterodimers form (termed S100A8/A9, or calprotectin) tetramers, a configuration that is vital for their biological activity (36). These proteins are mainly secreted by immune cells such as dendritic cells, neutrophils, monocytes, and activated macrophages (41).

These proteins are predominantly localized in the cytoplasm and relocate to the cytoskeleton and plasma membrane to increase intracellular calcium levels. Studies suggest that S100A8/A9 expression is downregulated during the maturation of monocytes to macrophages; its expression is also observed in macrophages present in inflamed tissues (36). Moreover, S100A8/A9 expression is seen in activated epithelial cells, endothelial cells, and keratinocytes. In both dendritic and myeloid-derived suppressor cells, S100A8/A9 protein expression is observed, with noticeably upregulated expression seen in dendritic cells treated with IL-10 (35). Fibrocytes are another population of S100A8/A9-expressing cells that are derived from myeloid lineage cells, which have been found to play a strong role in tissue repair and fibrosis. Upon exposure to the gram-negative bacterium *Porphyromonas gingivalis*, the S100A8/A9 protein complex can be induced in vascular smooth muscle cells, suggesting that this bacterium can trigger an inflammatory response within the blood vessel walls by activating S100A8/A9 in these cells (36).

### 2.3 Damage-associated molecular patterns

DAMPs are molecules within cells that are components of the innate immune response. They are released during cellular damage or cell apoptosis and tissue damage caused by trauma or pathogen infection (42). DAMPs cannot be recognized by the immune system under normal physiological conditions (43, 44). DAMP was first described by Seong and Matzinger in 2004 (45). DAMPs largely depend on the type of cells, such as epithelial or mesenchymal and injured tissue; however, DAMPs all share the common feature of stimulating an innate immune response within an organism (46). DAMPs serve as crucial mediators that link sterile inflammation to end-organ damage and life-threatening disease through the modulation of the innate immune response (11, 46).

Many of these DAMPs are intracellular proteins such as S100 proteins and HMGB1. Studies suggest that the HMGB1 protein has dual functions as a nonhistone nucleoprotein and an extracellular inflammatory cytokine. Intracellular HMGB1 is extensively bound to DNA and involved in transcriptional regulation, DNA replication and repair, telomere maintenance, and nucleosome assembly (47, 48). HMGB1 is prone to bind other proinflammatory molecules including DNA, RNA, histones, nucleosomes, lipopolysaccharide (LPS), SDF-1, IL-1α, IL-1β, and additional factors. These complexes act in synergy via cognate receptors to the HMGB1-partner molecules (47, 48). During injury or pathogenetic infection, HMGB1 is released and promotes inflammation. During this process, HMGB1 is passively released by necrotic but not apoptotic death of normal cells and actively secreted by a variety of activated immune and nonimmune cells (36, 47, 48).

However, some studies have demonstrated that HMGB1 is not a pro-inflammatory cytokine *per se* (47–49). HMGB1 by itself has little or no pro-inflammatory activity, but it binds to mediators of inflammation such as LPS, DNA, or IL-1β and induces signaling pathways leading to NF-κB activation, thereby potentiating inflammatory responses (50). Although the signaling pathways elicited by HMGB1 are not fully defined, there are studies suggesting that the triggering occurs via several receptors including the multiligand RAGE, TLR2, and TLR4 (51).

Likely, S100 proteins act both as intracellular mediators and as extracellular signaling proteins, which are able to regulate activities of the target cells in either a paracrine or autocrine manner. As intracellular calcium-binding molecules, S100A8/A9 have a role in migration and cytoskeletal metabolism. Cell damage or activation of phagocytes triggers their release into the extracellular space where they become danger signals that activate immune cells and vascular endothelium. S100A8/A9 seem to interact with RAGE4 and TLRs (52). Another member of S100 protein family, S100A12, is also found at high concentrations in inflamed tissue, where neutrophils and monocytes belong to the most abundant cell types. Even though S100A12 binds to RAGE, at least part of the proinflammatory effects of the S100A8/A9 complex depend upon interaction with other receptors (12).

DAMPs are associated with various diseases, including osteoarthritis (OA), neurodegenerative diseases, cancer, autoimmune diseases, and cardiovascular diseases (53). OA has been regarded as a degenerative joint disease that is characterized by the destruction of cartilage. Physical trauma and obesity are considered risk factors for OA (54). However, there is evidence suggesting that DAMPs-induced inflammation plays a crucial role, where S100 proteins are involved in the pathogenesis of OA (54). S100A8/A9 protein expression was elevated in the synovium of a collagenase-induced OA mouse model (54). Although S100A12 expression was unchanged in the serum between the OA patients and the healthy controls, the S100A12 level in the synovial fluid of OA patients was significantly increased in comparison to the healthy controls (55). Additionally, S100A12 escalated the secretion of MMP-13 and vascular endothelial growth factor in human OA chondrocytes, which suggests that S100A12 has a role in the progression of OA (56).

DAMPs are known to be associated with neuroinflammation in neurodegenerative disorders, including Alzheimer’s disease (AD) and Parkinson’s disease (PD) (57, 58). In AD patients, the levels of HMGB1 and soluble RAGE are significantly increased, which correlates with the levels of amyloid beta (59). A recent report demonstrated that HMGB1 and thrombin are triggers of inflammation and dysfunction of the blood-brain barrier (59). In AD patients, the serum levels of S100B were intimately related to the severity of the disease (60). The administration of pentamidine, an S100B inhibitor, reduced the levels of S100B and RAGE, thereby inhibiting neuroinflammation in the brain of an AD mouse model. Moreover, the role of the HMGB1-TLR4 axis is essential in the pathogenesis of PD. It has been demonstrated that the serum HMGB1 and TLR4 protein levels were significantly elevated in PD patients and correlated with the PD stages (61). Additionally, the S100B protein level was increased in the substantia nigra and cerebrospinal fluid of PD patients, and S100B was increased as well in the ventral midbrain of a mouse model treated with 1-methyl-4-phenyl-1,2,3,6-tetrahydropyridine (62).

The role of DAMPs in the pathogenesis of cancer is controversial. However, research suggests that DAMPs may mediate tumor progression by inducing chronic inflammation, which is a compound risk factor for tumor progression (63–65). Various studies demonstrated that DAMPs such as HMGB1 and S100 proteins activate inflammatory pathways and release promoters of carcinogenesis such as IL-1, IL-6, lymphotoxin (LT) -β, IFN-γ, TNF, and transforming growth factor (TGF)-β, which suggests DAMPs has a role in the development of early-stage carcinogenesis (63, 64, 66).

The involvement of DAMPs with rheumatoid arthritis has been demonstrated in various studies (67, 68). These studies suggest that upregulated S100A8/A9 and S100A11/A12 proteins were observed in the synovial tissue, synovial fluid, or serum of rheumatoid arthritis patients (69, 70). In addition, the expression of HMGB1 was elevated in the serum and synovial fluid of rheumatoid arthritis patients in a number of studies (47, 71).

Involvement of S100 proteins in the pathogenesis of atherosclerosis indicating a significant role of DAMPs in cardiovascular diseases (72). Atherosclerosis is an inflammatory disease of the arterial wall, in which the vessels narrow due to accumulating plaques of inflammatory cells and lipids (73). It has been demonstrated that S100A8/A9 exist in plaques, and they increase atherogenesis by activating neutrophils and monocytes in arterial lesions (72). Moreover, S100A8/A9 and S100A12 have a crucial role in the mediation of inflammation and increase atherosclerosis in human and rodent models by interacting with RAGE, which plays an important role in endothelial dysfunction and inflammation (21). In addition, it has been reported that, among conventional risk factors, S100A12 showed the strongest association with the risk of coronary heart disease (74). DAMPs may also have a role in pulmonary hypertension, which is characterized by perivascular infiltration of inflammatory cells and pulmonary vascular remodeling, ultimately resulting in the right heart failure and premature death (75).

During cellular stress or tissue damage, released DAMPs can be detected by multiple pattern recognition receptors. NLRP3 inflammasome is a cytosolic pattern recognition receptor that is commonly associated with the immune response to bacteria, viruses, fungi, and parasites (5). In most cases, the recognition of pathogens in the immune response is indirect. TLRs recognize the particular components of the invader and then induce the NLRP3 inflammasome components to be transcribed and assembled (76). Upon activation by cellular damage or tissue stress, NLRP3 triggers the formation of the inflammasome, also known as a multiprotein signaling complex, that activates caspase-1, leads to the secretion of IL-1β and IL-18, and induces pyroptosis, a form of cell death that is a major pathway of inflammation (5). Studies demonstrate a close association to various inflammatory diseases, including type 2 diabetes, gout, neurodegenerative diseases, and pulmonary hypertension (77).

Growing evidence suggests that two DAMPs, S100A8 and S100A9, play a significant role in activating the NLRP3 inflammasome (13, 29). They are involved in inflammation caused by stress, injury, and microorganisms. After being affected, neutrophils, macrophages, and monocytes intensely express and secrete S100A8/A9 to modulate inflammatory processes with the induction of inflammatory cytokines, ROS, and nitric oxide (13, 38, 39). The released S100A8/A9 proteins can promote IL-1β secretion in neutrophils and macrophages in an autocrine or paracrine fashion, as well as prime the NLRP3 inflammasome (29, 78). Studies have shown that S100A8/A9 proteins activate NLPR3 inflammasome signaling to promote the pathogenesis of several diseases, including myelodysplastic syndromes and airway obstructive diseases (13, 41). In one study, a close correlation between S100A8 and pyroptosis was observed, and a direct effect of S100A8 on macrophage pyroptosis was identified (13). This study also demonstrated that S100A8 interacts with the TLR4 and then activates downstream Nm NF-κB with transcriptional upregulation of NLRP3, pro-IL-1β, and pro-IL-18 (13). ROS has been reported as the second signal for NLRP3 activation, playing a crucial role in fibrotic progression (13, 20, 79). A number of studies suggest that S100A8/A9 have an immunoregulatory role as intracellular differentiation markers. After secretion to the extracellular compartment, they also function as innate amplifiers of inflammation in various inflammatory diseases via two receptors, TLR4 and RAGE, which leads to increased production of pro-inflammatory cytokines like IL-1β and contributes to various inflammatory diseases including pulmonary hypertension (20, 79, 80).

## 3 Regulatory role of S100A8/A9 in NLRP3 inflammasome

During inflammation, S100A8/A9 is released vigorously and exerts a critical role in modulating the inflammatory response by activating NF-κB1 through ROS-dependent activation (80). The calcium binding proteins S100A8/A9 serve as a candidate biomarker for diagnosis and follow-up, and they have a role as a predictive indicator of therapeutic responses to inflammation-associated diseases (80).

### 3.1 Function of NLRP3 inflammasome

NLRP3 is an intracellular receptor that senses foreign pathogens and self-danger signals, leading to the formation and activation of the NLRP3 inflammasome (29). NLRP3 inflammasome is an important component of the innate immune system’s response to pathogens, which consists of a set of cytoplasmic multiprotein complexes (5). Different inflammasomes have distinct stimulatory signals but have very conserved downstream effects, especially in the activation of caspase-1, which in turn triggers the three key substances: IL-1β, pro-IL-18, and gasdermin D (GSDMD) (3, 4).

**Figure 3:**
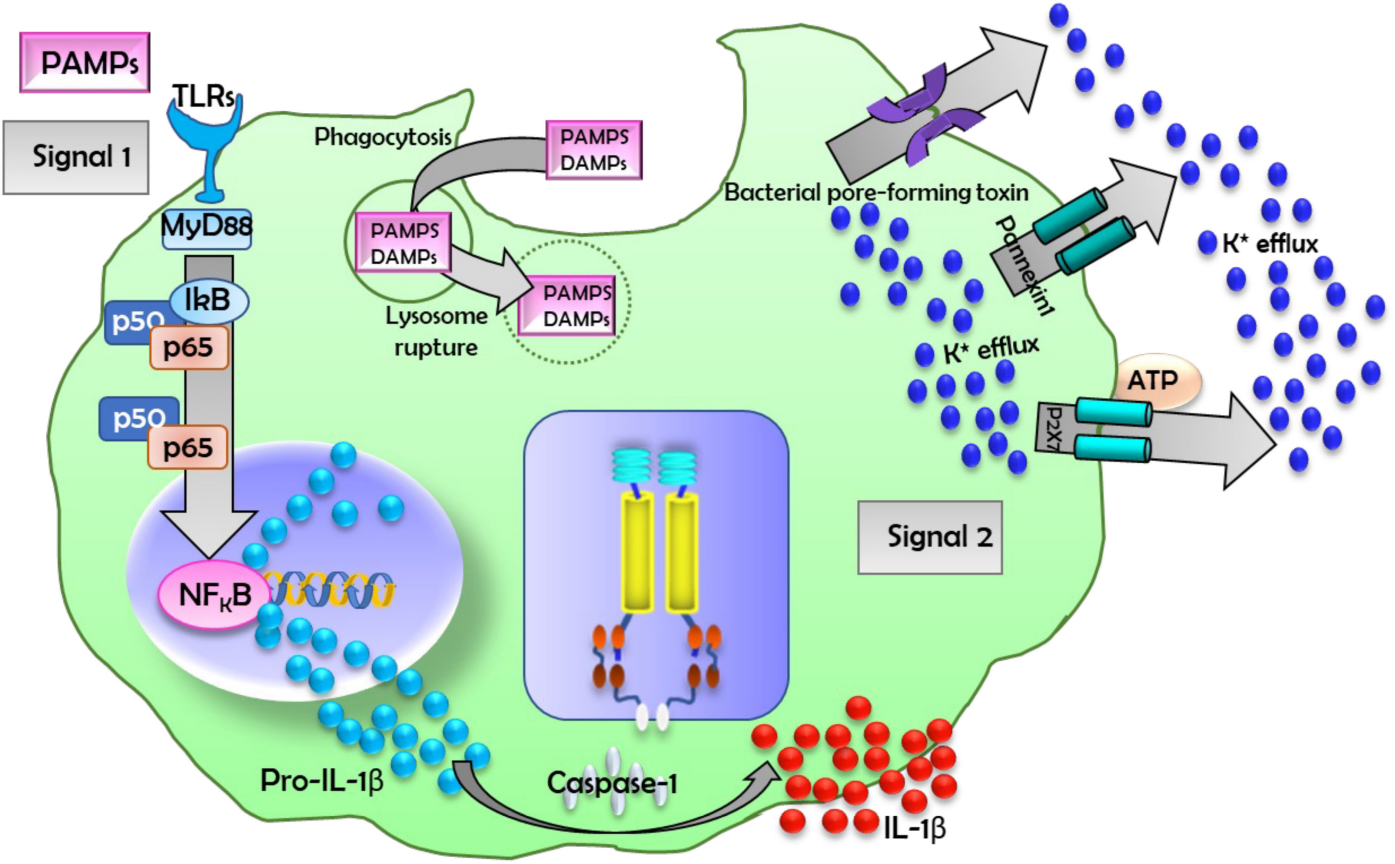
A schematic diagram for NLR-related protein 3 (NLRP3) inflammasome activation. A number of stimuli trigger the activation of NLRP3, which secretes interleukin (IL)-1β and IL-18. Diverse pathogen-associated molecular patterns (PAMP) and/or DAMP stimulation potentiate two signals that activate the NLRP3 inflammasome. Signal 1 activation leads to the expression of the pro-IL-1β gene and the production of the pro-IL-1β protein through the toll-like receptor (TLR)-MyD88-NFκB signaling pathway. Signal 2 is a critical step in inflammasome activation. These signals or agonists trigger the assembly of a large macromolecular complex through the recruitment of the apoptosis-associated speck-like protein containing a C-terminal caspase-recruitment domain adaptor protein and pro-caspase-1 to NLRP3. Several mechanisms have been suggested for NLRP3 inflammasome activation, including pore formation through P2X7 receptor and K+ efflux, mitochondrial reactive oxygen species generation, phagocytic pathway activation by particulate or crystalline structures (e.g., monosodium urate crystals, aluminum potassium sulfate, or silica nanoparticles), and lysosome rupture. The molecular mechanisms by which NLRP3 inflammasome activation occurs are not yet fully understood. ATP, adenosine triphosphate.

The NLRP3 protein belongs to the family of nucleotide-binding oligomerization domain-like receptors (NLRs), and NLRP3 protein contains a leucine-rich repeat domain at the carboxyl terminus, a pyrin domain at the amino terminus, and a nucleotide-binding domain in the center domain (5). It has been reported that the NLRP3 inflammasome can rapidly and effectively eliminate microbial infection and repair damaged tissue (3, 5). In a number of studies, DAMP molecules S100A8/A9 were shown to be actively involved in the inflammatory response by regulating neutrophil functions (9). S100A8/A9 induce the secretion of several pro-inflammatory cytokines in monocytes, including IL-6, TNFα, and IL-1β, through stimulating the production of ROS (9, 72). This phenomenon activates the transcription factor NF-kB, which leads to cytokine secretion and expression and results in the activation of the NLRP3 inflammasome (29, 81). NLRP3 activation and function is shown in **Figure 3**.

### 3.2 S100A8/A9 in the priming and activation of the NLRP3 inflammasome

**Figure 4:**
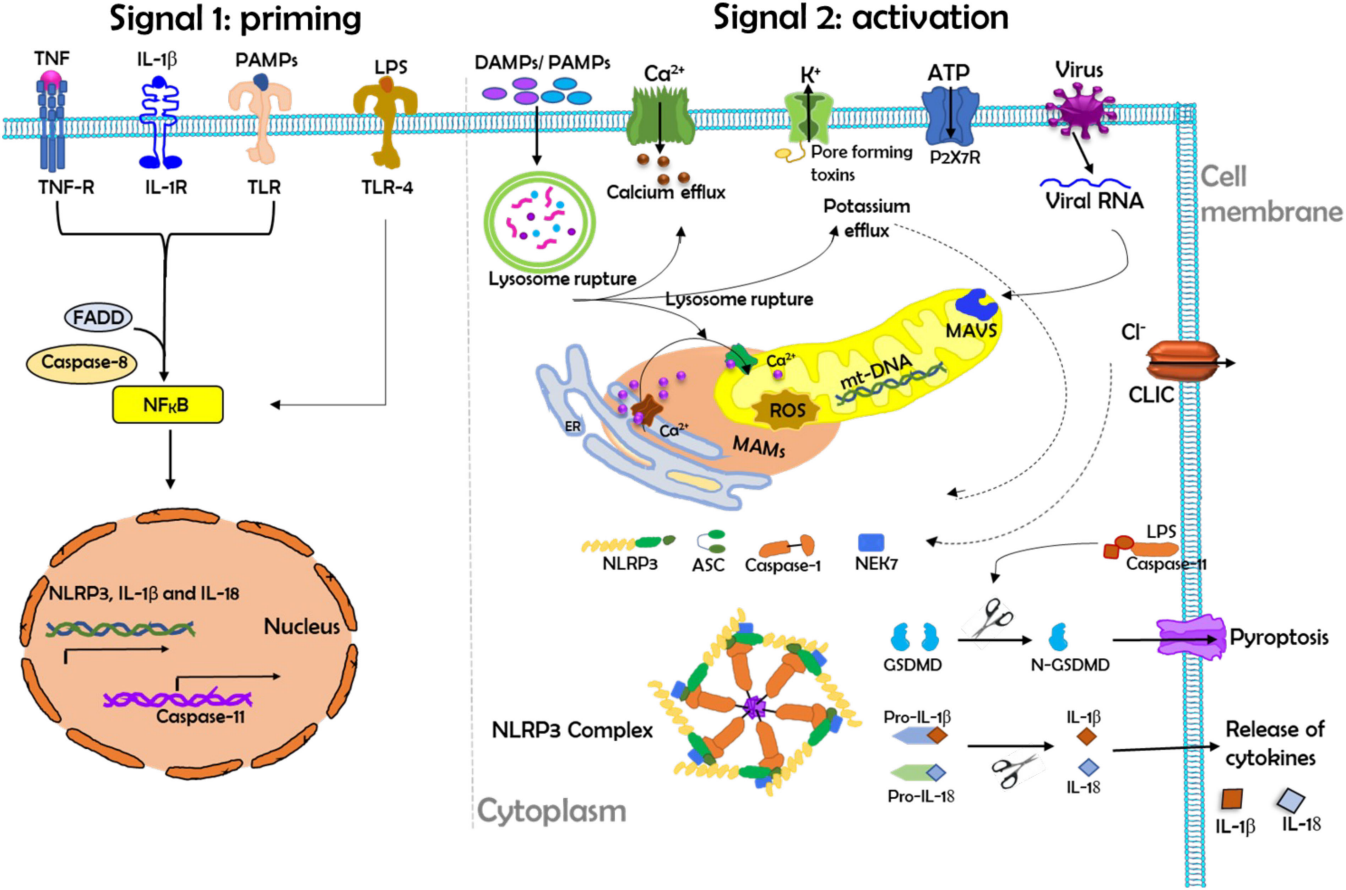
The schematic diagram demonstrates the priming and activation of the NLRP3 inflammasome. Herein, the activation process of the NLRP3 inflammasome requires two main signals. Left: Signal 1 (priming) leads to the activation of the transcription factor NF-κB and the subsequent transcription of canonical and noncanonical NLRP3 inflammasome components. Priming is provided by exposure to pathogen-associated molecular patterns (PAMPs) such as LPS or by endogenous cytokines that activate receptors at the cell membrane. During priming, the induction of NLRP3 expression is controlled by FAS-associated death domain protein (FADD) and caspase-8. Right: Signal 2 (activation) is responsible for NLRP3 complex assembly and the subsequent release of inflammatory cytokines (IL-1β and IL-18). NLRP3 activation is provided by a plethora of stimuli, such as PAMPs or DAMPs, ATP, and viral RNA. NLRP3 activation in turn triggers downstream signaling events such as mitochondrial damage, mitochondrial ROS production, lysosomal disruption, and ion (K^+^ and Ca^2+^) efflux. During activation, mitochondrial antiviral signaling protein (MAVS) mediates the NLRP3 activation induced by RNA viruses, where excessive Ca^2+^ released from the ER causes mitochondrial dysfunction and is implicated in NLRP3 inflammasome activation. Chloride intracellular channel protein (CLIC)-mediated Cl^-^ efflux promotes the NEK7-NLRP3 interaction and subsequent NLRP3 inflammasome assembly. Herein, LPS can directly activate TLR4 to induce the transcription and activation of caspase-11, which in turn cleaves the pore-forming protein gasdermin D (GSDMD), which can induce pyroptosis. IL-18, interleukin-18; IL-1β, interleukin-1beta; IL-1R, interleukin 1 receptor; mt-DNA, mitochondrial DNA; ROS, reactive oxygen species; TLR, Toll-like receptor.

The NLRP3 inflammasome is a multiprotein complex that is a critical component of the innate immune system. NLRP3 inflammasome consists of a sensor NLRP3; it is an NLR family protein containing a pyrin domain 3, an adaptor, apoptosis-associated speck-like protein, which contains a caspase activation and recruitment domain and an effector called caspase-1 (82). This tripartite protein comprises three domains: an amino-terminal pyrin domain, a central NACHT domain (domain present in NAIP, CIITA, HETE, and TP1), and a carboxy-terminal leucine-rich repeat domain (82, 83). It has been demonstrated that the activation process of NLRP3 inflammasome depends upon two signals: a priming signal that is required for the upregulation of NLRP3 and pro-IL-1β and a second signal that triggers assembly into the NLRP3 inflammasome complex shown in **Figure 4** (82). These two signals have been proposed to underlie NLRP3 activation: priming and activation (**Figure 4**).

The priming signal is triggered by the engagement of TLRs by their ligands or endogenous molecules, such as TNF or IL-β, which then leads to the transcription of NLRP3 and pro-IL-1β via the regulation of NF-κB (84). Recently, it has been shown that the induction of NLRP3 expression during priming is also controlled by FAS-associated death domain protein and caspase-8 (83). During the activation signal, NLRP3 responds to many stimuli for activation of the inflammasome. A variety of pathogen-associated molecular patterns (PAMPs) and DAMPs provide the activation signal for NLRP3. The PAMPs and DAMPs originated from numerous pathogens, a large number of pore-forming toxins, adenosine triphosphate, and particulate crystals and aggregates (76, 82, 85).

Recent studies have demonstrated that cell stress in NLRP3 inflammasome-associated autoinflammatory disease enhanced ATP release and maintained high levels of IL-1β and IL-18 in blood monocytes (82). Moreover, ATP-induced NLRP3 inflammasome activation is differentially regulated between dendritic cells and macrophages (82). As observed in the dendritic cells, stimulation with TLR ligands in the absence of ATP was sufficient to produce mature IL-1β (86). Increasing evidence suggests a regulatory role of S100A8/A9 in the NLRP3 inflammasome. For example, S100A8/A9 are capable of promoting the expression of pro-inflammatory mediators, including TNF-a, IL-1β, IL-6, and IL-8, through NF-kB activation in THP-1 cell line and human macrophages (87). Additionally, S100A8/A9 have a regulatory role in NLRP3 inflammasome, as these proteins influence the redox balance in human monocytes (87). The mechanism of enhanced ROS production is still unclear. However, evidence suggested mediating effects on ROS production via TLR4 activation (87).

It has been proposed that intracellular signals induced by S100A8/A9 are likely to be similar to LPS (87). LPS is known to trigger ROS production, which is crucial for the expression of inflammatory cytokines through the activation of redox-sensitive transcription factors (87). LPS stimulates the NF-kB-driven protein synthesis that primes the NLRP3 inflammasome, which greatly potentiates the release of mature IL-1β (87). However, several studies have shown that prolonged stimulation of monocytes without a co-signal like ATP leads to a slow activation of caspase-1 and the release of IL-1β (87–90). On their own, S100A8/A9 induce IL-1β secretion after 24 h of stimulation; however, the timeframe is greatly reduced by the addition of ATP (87). One study reported that 4 h of priming with S100A8/A9 and 30 min of stimulation with ATP were sufficient to observe caspase-1 cleavage and IL-1β release (87). It demonstrates the ROS- and NF-κB-mediated NLRP3 inflammasome priming by S100A8/A9 in human peripheral blood mononuclear cells, suggesting that S100A8/A9 plays a crucial role in redox-sensitive biological responses (87). In another study, Prunenster et al. demonstrated a governing role of S100A8/A9 in NLRP3 inflammasome induced by E-selection (13). This cell adhesion molecule is induced in endothelial cells by inflammatory stimulation, injury, or other factors. Recently, Liu Yan et al. reported a role of S100A8 as a stimulator NLRP3 inflammasome dependent pyroptosis in macrophages via activating TLR4/NF-κB signaling and inducing ROS abundance, which leads to the progression of liver fibrosis (13). Additionally, a review article by David A. Sallman et al. described how S100A9 trigger the pyroptosis through the generation of reactive oxygen species, which leads to assembly and activation of the redox-sensitive NLRP3 inflammasome, assuring propagation of the myelodysplastic syndromes (91).

### 3.3 DAMP-NLPR3 inflammasome signaling axis in autoimmune diseases

Multiple studies have shown that overactivation of the NLRP3 inflammasome results in excessive inflammation and tissue damage, leading to pathological conditions that contribute to autoimmune diseases (92). Mechanistically, the NLRP3 inflammasome serves as a key checkpoint in both innate and adaptive immunity during the development of autoimmune diseases. Early immune responses can be triggered by immune dysfunction, often due to the erroneous targeting of self-antigens, which is frequently associated with heightened immune activity (92, 93). Damaged tissues and cells release DAMP molecules, which act as alarm signals (94). Interestingly, direct binding of stimuli to NLRP3 is rarely observed (92), suggesting that NLRP3 detects common upstream signals induced by inflammasome activators. This mode of indirect activation is fundamental to understanding how NLRP3 functions at a molecular level. The interaction between NLRP3 inflammasome and DAMP signaling is central to elucidating this mechanism. DAMP signaling can initiate priming via NF-κB, leading to the increased expression of inflammasome components, including NLRP3, pro-caspase-1, pro-IL-18, and pro-IL-1β. After priming, NLRP3 assembly results in full inflammasome activation, culminating in caspase-1-dependent cleavage and activation of IL-1β and IL-18 (95). As a DAMP player, HMGB1 has been identified as a regulator of the NLRP3 inflammasome, indicating that NLRP3 inflammasome interacts with S100A8 and S100A9, as these two DAMP molecules share the same receptors as HMGB1, including RAGE and TLR4 (96).

DAMP molecules amplify and sustain injury-induced inflammatory responses via autocrine and paracrine mechanisms, primarily through RAGE-dependent pathways (97). This process also facilitates post-inflammatory fibrotic repair and vascular remodeling in injured tissues (98, 99). Specifically, S100A8 and S100A9 regulate the NLRP3 inflammasome through RAGE, TLR4, and NF-κB signaling pathways (87). These two S100 molecules are crucial neutrophil-derived proteins that are highly conserved between humans and mice (100–102). They have been identified as potent inducers of the pro-inflammatory phenotype in macrophages (36, 103). Consequently, through regulation of innate immune cells—particularly neutrophils and macrophages—activation of DAMP-inflammasome signaling acts as a pivotal component of the innate immune response to various stimuli, including self-antigens, thereby contributing to the onset and progression of autoimmune diseases.

Adaptive immunity extends from innate immunity (5, 93). Innate immune responses initiate the adaptive immune process, enabling the host to develop long-lasting, effective defenses (5, 93). DAMP-inflammasome signaling would play a crucial role in regulating adaptive immune responses in autoimmune diseases (93). Cytokines produced by the NLRP3 inflammasome, specifically IL-1β and IL-18 (93), promote the differentiation of naive T cells into effector and memory T cells, thereby activating adaptive immunity (3, 92). By driving both innate and adaptive immune responses, the NLRP3 inflammasome is linked to the pathogenesis of various autoimmune diseases, including type 1 diabetes, cystic fibrosis, rheumatoid arthritis, autoimmune colitis, psoriasis, systemic lupus erythematosus, and systemic sclerosis (4, 93). Elevated levels of S100A8 and S100A9 proteins, released from activated phagocytes in patients with the above-listed autoimmune disorders, serve as biomarkers for diagnosis and prognosis (104). Notably, diseases like psoriasis and arthritis exhibit patchy inflammation despite systemic immune dysregulation (93), indicating that localized DAMPs are essential for inflammatory manifestations at the sites. Conversely, systemic autoimmune conditions such as systemic sclerosis can progress to organ-specific diseases like pulmonary hypertension (105). Studies have shown that activation of DAMP signaling pathways, particularly HMGB1-RAGE, triggers macrophage activation, pulmonary endothelial cell apoptosis, and vascular smooth muscle cell proliferation, mechanisms central to pulmonary vascular remodeling post-injury (98, 99, 106, 107). As RAGE ligands, S100A8/A9 would be critical in regulating inflammasome pathways in pulmonary hypertension, a topic further elaborated below. Prior research indicates positive feedback loops mediated by DAMP signaling including TLRs, RAGE, and NF-κB (97–99, 106, 107), which are crucial for transitioning from acute to chronic immune responses as well as for switching immune cells from a pro-inflammatory to a pro-proliferative phenotype (99). These mechanisms facilitate the progression from innate to adaptive immunity, contribute to inflammation-driven fibrosis, and highlight the pivotal role of the DAMP-inflammasome signaling axis in orchestrating this transition.

### 3.4 Contribution of 100A8/A9 and NLRP3 in pulmonary hypertension

Pulmonary hypertension remains one of the most challenging entities to diagnose and treat due to the subtlety and nonspecificity of its symptoms and signs, the lack of availability of sensitive, noninvasive, accurate diagnostic tests (108). There is increasing evidence suggesting the association of systemic autoimmune diseases with pulmonary hypertension (108). Various studies have demonstrated the role of S100A8/A9 in promoting inflammation, fibroblast growth, and collagen production in various lung diseases including pulmonary hypertension. S100A8/A9 is a proinflammatory factor that activates the complement factor system to amplify the inflammatory response. For example, Guo et al. have demonstrated a correlation between S100A8/A9 and sepsis-induced lung damage (109). They have reported that S100A8/A9 expression significantly increased in the lungs of cecal ligation and puncture (CLP) operated mice (109). The knockout (KO) of S100A8/A9 noticeably mitigated pulmonary inflammation, vascular leakage, and acute lung injury, resulting in improved survival outcomes in septic mice (109). In another study, Du et al. demonstrated a role of S100A8/A9 as an initial proinflammatory factor that triggers cardiac fibroblasts activation, thus amplifying the inflammatory response and initiating cardiac damage (110).

NLPR3 inflammasomes are multi-protein complexes involved in sensing both endogenous and exogenous cellular stress. They are triggered by DAPMs and PAMPs based on the identity of pattern recognition receptor within the complex (2). The formation of the NLRP3 inflammasome complex is beneficial to the proximity-induced autocatalytic activation of pro-caspase-1 to cleaved caspase-1. In this process, the role of caspase-1 downstream is the cleavage of cytokines such as pro-IL-1β and pro-IL-18 to their biologically active forms. Herein, caspase-1 additionally cleaves GSDMD (111). GSDMD is a protein in which the N-terminal subunits assemble into a multi-unit complex, forming a pore in the plasma membrane that acts as a key executioner in the release of active IL-1β and induction of pyroptosis (112). A number of studies have demonstrated the association of NLRP3 inflammasome and GSDMD in pulmonary hypertension. Both NLRP3 and GSDMD have been found playing a role in pulmonary hypertension by activating the proinflammatory cytokines and releasing cytosolic contents into the extracellular space (111, 113). In particular, GSDMD mediated pyroptosis plays major role in endothelial dysfunction, which leading to pulmonary hypertension (112).

Our ongoing research has also indicated the relation of DAMP molecules and NLRP3 inflammasome in inducing pulmonary hypertension, which is consistent with other studies (85, 111). DAMP molecules regulate NLRP3 inflammasome through the receptors RAGE and TLR4 (87), which then triggers a signaling cascade involving NF-κB transcription factor, ultimately leading to the activation of the NLRP3 inflammasome complex and the secretion of GSDMD, causing cell death through pyroptosis (114). DAMPs such as S100A8/A9 are reported as crucial components for inducing NLRP3 inflammasome priming. S100A8/A9 are proinflammatory mediators released by myeloid cells during many acute and chronic inflammatory disorders, suggesting a regulatory role in NLRP3 inflammasome (13, 29, 83). However, the contribution of NLRP3 inflammasome, S100A8/A9, and GSDMD as a team to pulmonary hypertension is unclear. Understanding the interactive and feedback mechanism between NLRP3 inflammasome, S100A8/A9, and GSDMD is necessary to explore their role in pulmonary hypertension. Moreover, finding the precise mechanism of how to maintain regulated NLRP3 activation might be the key to control various autoimmune diseases including pulmonary hypertension, which has yet to be investigated.

## 4 Conclusion

In this review, we summarized the immunoregulatory mechanisms by which S100 family proteins regulate the inflammasome signaling axis. A fundamental commonality among S100 family proteins is their engagement of the DAMP–NLRP3 pathway. Many members—including S100A8/A9, S100A12, and S100B—interact with DAMP receptors such as RAGE and TLR4, leading to NF-κB activation, ROS generation, and subsequent inflammasome priming (12, 20, 29, 87, 95). These shared pathways enable S100 family proteins to amplify inflammatory responses across diverse pathological contexts.

Within this family, S100A8 and S100A9 possess distinctive regulatory features. They are among the most abundantly expressed DAMPs in neutrophils and monocytes (29, 38), constituting up to 40% of neutrophil cytosolic protein and approximately 5% in monocytes (39, 115). With such high expression and potent immunoregulatory properties, they can influence both the priming and activation phases of NLRP3 inflammasome assembly (29, 87, 95). Notably, S100A8/A9 can synergize with secondary signals such as ATP to accelerate IL-1β maturation (29, 87), promote pyroptosis via GSDMD activation (13, 91), and sustain inflammatory loops in chronic diseases. The dual role of S100A8/A9 as potent inflammatory mediators, DAMP molecules, and sensitive biomarkers, combined with their strong translational relevance in conditions such as pulmonary hypertension and autoimmune diseases (87, 98, 99, 105, 106), underpins our emphasis on these proteins as the primary focus of this review. At the same time, we recognize the important contributions of other S100 family members, which are summarized in **Table 1**, and highlight the need for further studies to delineate their individual and overlapping roles, as well as the interactive mechanisms between different S100 proteins in NLRP3 inflammasome regulation.

S100A8 and S100A9 levels are markedly increased in a wide range of inflammatory conditions, including autoimmune diseases, cancers, and neurodegenerative disorders, and they may serve as effective biomarkers for disease detection and prognosis (116). As key DAMPs, these proteins are crucial in host anti-infective immunity. Excessive activation of the NLRP3 inflammasome is linked to the development of various autoimmune diseases, underscoring the importance of maintaining its activity within a regulated range. Further investigation is needed to elucidate the mechanistic relationships between NLRP3 inflammasome signaling and DAMPs in diverse autoimmune diseases. In particular, dissecting the feedback processes between the NLRP3 inflammasome, S100A8/A9, and GSDMD could be a crucial step in understanding their roles in inflammatory pathology. Such insights may ultimately facilitate the development of strategies to modulate the DAMP–NLRP3 axis and maintain balanced inflammasome activation, aiding in the diagnosis and treatment of autoimmune and inflammatory diseases, including pulmonary hypertension, which remains one of the most challenging conditions to diagnose and manage today.
